# Supplementary material for: Risk of Metachronous Neoplasia with High-Risk Adenoma and Synchronous Sessile Serrated Adenoma: A Systematic Review and Meta-Analysis
Source: Diagnostics (Basel). 2023 Apr 27;13(9):1569. doi: 10.3390/diagnostics13091569 (PMC10177994; doi:10.3390/diagnostics13091569)
Supplement: Supplementary file 1 [file diagnostics-13-01569-s001.zip › Supplementary Table S1.pdf]

Supplementary Table S1. Risk of Bias Assessment Tool for Non-Randomized Studies (RoBANS) of all included studies

Study	Selection		Performance	Detection	Attrition	Reporting
	Selection of Participants	Confounding variables	Measurement of exposure	Blinding of outcome assessment	Incomplete outcome data	Selective outcome reporting
**Anderson et al**	Unclear	Low	Low	Unclear	Low	Low
**Macaron et al**	Low	Low	Low	Unclear	Low	Low
**Melson**	Unclear	Low	Low	Unclear	Low	Low
**Park et al.**	Unclear	Low	Low	Unclear	Low	Low
**Pereyra et al**	Unclear	low	Low	Unclear	Low	Low
**Schreiner**	Unclear	Low	Low	Unclear	Low	Unclear
