# Supplementary figures and images for: Risk of Metachronous Neoplasia with High-Risk Adenoma and Synchronous Sessile Serrated Adenoma: A Systematic Review and Meta-Analysis
Source: Diagnostics (Basel). 2023 Apr 27;13(9):1569. doi: 10.3390/diagnostics13091569 (PMC10177994; doi:10.3390/diagnostics13091569)

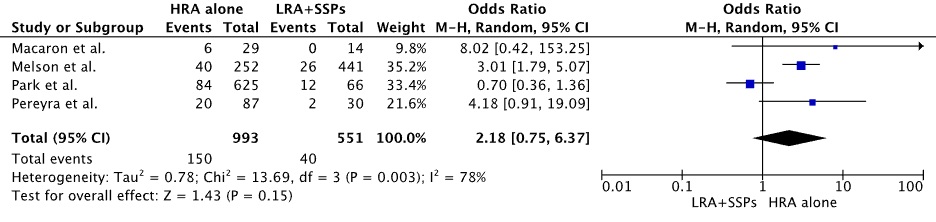

Supplement: Supplementary file 1 [file diagnostics-13-01569-s001.zip › Supplementary Figure S1.jpg]
